# Linking planar polarity signalling to actomyosin contractility during vertebrate neurulation

**DOI:** 10.1098/rsob.240251

**Published:** 2024-11-20

**Authors:** Sarka Novotna, Lorena Agostini Maia, Katarzyna Anna Radaszkiewicz, Pavel Roudnicky, Jakub Harnos

**Affiliations:** ^1^ Department of Experimental Biology, Faculty of Science, Masaryk University, Brno 62500, Czechia; ^2^ CEITEC-Central European Institute of Technology, Masaryk University, Brno 62500, Czechia; ^3^ National Centre for Biomolecular Research, Faculty of Science, Masaryk University, Brno 62500, Czechia

**Keywords:** actomyosin contractility, planar cell polarity, vertebrates, neurulation, *Xenopus *embryos, MDCK cells

## Abstract

Actomyosin contractility represents an ancient feature of eukaryotic cells participating in many developmental and homeostasis events, including tissue morphogenesis, muscle contraction and cell migration, with dysregulation implicated in various pathological conditions, such as cancer. At the molecular level, actomyosin comprises actin bundles and myosin motor proteins that are sensitive to posttranslational modifications like phosphorylation. While the molecular components of actomyosin are well understood, the coordination of contractility by extracellular and intracellular signals, particularly from cellular signalling pathways, remains incompletely elucidated. This study focuses on WNT/planar cell polarity (PCP) signalling, previously associated with actomyosin contractility during vertebrate neurulation. Our investigation reveals that the main cytoplasmic PCP proteins, Prickle and Dishevelled, interact with key actomyosin components such as myosin light chain 9 (MLC9), leading to its phosphorylation and localized activation. Using proteomics and microscopy approaches, we demonstrate that both PCP proteins actively control actomyosin contractility through Rap1 small GTPases in relevant *in vitro* and *in vivo* models. These findings unveil a novel mechanism of how PCP signalling regulates actomyosin contractility through MLC9 and Rap1 that is relevant to vertebrate neurulation.

## 1. Introduction

Actomyosin-mediated contractility is a conserved mechanism to generate mechanical force in living cells underlying cellular motility and tissue morphogenesis. While actomyosin-mediated contractility in striated and smooth muscle cells is well understood, the regulation of such contractility by signalling pathways in non-muscle cells remains less understood [1] (electronic supplementary material, figure 1A). At the molecular level, actomyosin contractility relies on non-muscle myosin II, comprising two heavy chains, essential light chains (ELCs) and regulatory light chains (RLCs, or further denoted as MLCs). Phosphorylation of MLC at Ser 19 (pMLC) [2–4] regulates the myosin II structural conformation and activity [5], which is crucial for generating contractile force [1].

**Figure 1.**
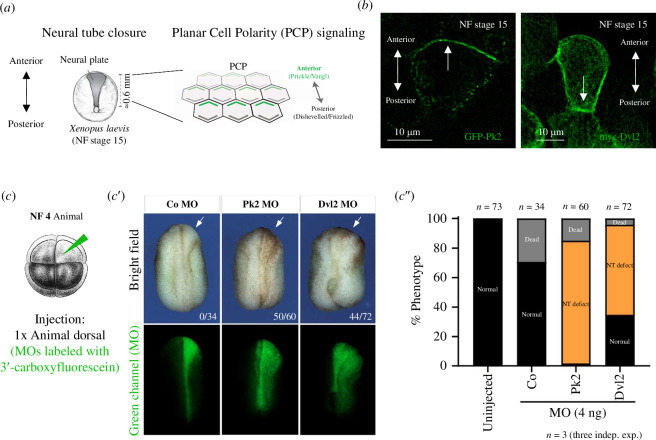
The role of PCP proteins during vertebrate neurulation, demonstrated using the *Xenopus* neural plate. (*a*) NT closure, a crucial developmental event in vertebrate embryos, exemplifies significant cell shape rearrangements. PCP, comprised of two protein complexes, is essential in this process. (*b*) In the *Xenopus* neural plate at NF stage 15, the PCP protein Pk2 is polarized anteriorly, while Dvl2 is polarized predominantly posteriorly. (*c*) *Xenopus* embryos were microinjected into one dorsal blastomere at the animal pole during the eight-cell stage. (*c'*) In *Xenopus* embryos with Pk2 and Dvl2 knockdown via morpholino injection into one dorsal blastomere, defects in NT closure are visible in the bright field. The morpholino is detectable in the green channel below. The numbers in the bottom right corner correspond to the amount of embryos with NT defects versus the total amount. White arrows point to the injected side of embryos. (*c″*) Phenotypic graph of morpholino-injected *Xenopus* embryos. Most embryos injected with Pk2 morpholino and a significant portion of embryos with Dvl2 morpholino exhibited NT defects, highlighting the key role of both proteins during vertebrate neurulation.

Neural tube (NT) closure in vertebrates [6–8], which has fascinated and challenged scientists for more than 140 years [9], emerges as an exemplary *in vivo* model for studying actomyosin contractility. NT closure is a complex morphogenetic event driven by a set of coordinated cell movements and cell shape changes, all mediated by the actomyosin contractile apparatus [9–12]. Genetically, NT closure has been shown to be regulated by the planar cell polarity (PCP) signalling pathway (figure 1*a*) [13–16], representing a fundamental cueing system pervasive in multicellular organisms [17–23]. Yet, the precise mechanism underlying how PCP proteins interact with the actomyosin network to influence NT morphogenesis remains enigmatic.

We have learned from studies in both *Drosophila* and vertebrates that at the molecular level, PCP manifests through the spatial segregation of two distinct protein complexes within cells, the Vangl/Prickle (Vangl/Pk) complex anteriorly and the Frizzled/Dishevelled (Fzd/Dvl) complex posteriorly (figure 1*a*; for comprehensive reviews, see [12,17,18,20,23–25]). While the posterior PCP complex, comprising Dvl and its associated formin protein Daam1 [26–28] is postulated to activate RhoA GTPase and Rho-kinase [29,30], thus modulating myosin II activity, empirical validation of this model within an *in vivo* context remains lacking. Notably, the primary evidence supporting this model stems from studies conducted on tissue culture cells lacking PCP [28], wherein PCP proteins are not polarized—a limitation inherent to this *in vitro* system. Furthermore, it is crucial to acknowledge that this model solely addresses one side of polarized cells, focusing on the localization of the Fzd/Dvl complex. While this might be useful in the asymmetrical growth of bristles or trichomes in *Drosophila* [19,20], NT closure in vertebrates inherently requires coordination between both sides of the cell. The observed colocalization of Pk proteins and MLC and myosin II [31,32], along with the data presented in this study suggest a pressing need to reassess the prevailing model of actomyosin contractility in vertebrates.

To interrogate the *in vivo* function of PCP proteins in actomyosin contractility and vertebrate neurulation, we leverage the utility of *Xenopus* embryos, a well-established model system for studying morphogenesis. The advantages conferred by this model, including ample biochemical resources, precise protein expression targeting capabilities, external development of embryos and NT developmental similarities to mammals [12], underscore its suitability for dissecting NT processes in vertebrates. Crucially, the translational relevance of insights garnered from *Xenopus* studies to human embryogenesis, especially in the context of addressing NT closure defects, accentuates its significance [33,34]. Furthermore, the *Xenopus* neural ectoderm serves as an exemplary model for studying vertebrate planar polarized tissue [31,35] and has also been crucial for elucidating *in vivo* actomyosin contractility dynamics [36]. Using *Xenopus* embryos, we demonstrate the physical and functional interactions of anteriorly localized Pk2 and posteriorly polarized Dvl2 with actomyosin complexes, notably MLC9 (also known as Myl9), culminating in the localized inductions of pMLC via Rap1 GTPase and Rap1GAP2 in the *Xenopus* neural plate. These findings shed light onto the detailed mechanisms through which cytoplasmic PCP proteins together with their binding partner Casein kinase 1ε (CK1ε) modulate the actomyosin contractile network during vertebrate embryogenesis, underscoring their direct physiological relevance *in vivo*.

## 2. Results

### 2.1. MLC9, Pk2 and Dvl2 as candidates for PCP signalling and actomyosin network in the *Xenopus* neural plate

MLC9, Pk2 and Dvl2 were chosen as candidates for PCP signalling and the actomyosin network due to their localization in the *Xenopus* neural plate during critical developmental stages and their established roles in previous studies [28,31,37]. First, we examined the subcellular localization of Pk2 and Dvl2 in the *Xenopus* neural plate at NF (Nieuwkoop and Faber) stage 15. As anticipated, Pk2 was localized anteriorly, while Dvl2 was found posteriorly (figure 1*b*). However, in some cases, Dvl2 did not exhibit polarization (electronic supplementary material, figure 1B), perhaps due to its versatile functions in other cellular signalling pathways [38–41]. To elucidate the roles of endogenous Pk2 and Dvl2 proteins, we knocked down their mRNAs in the *Xenopus* neuroectoderm by injecting morpholino oligonucleotides labelled with 3′-carboxyfluorescein into one dorsal cell of the eight-cell stage embryos (figure 1*c*). By NF stage 17, the neural plate failed to close on the injected side for both morpholinos, whereas the opposite side closed properly (figure 1*c′*). Almost all embryos injected with Pk2 morpholino exhibited NT defects. Embryos injected with Dvl2 morpholino showed similar results, although around 30% of them maintained a normal phenotype (figure 1*c″*).

These results demonstrate that both cytoplasmic PCP proteins, particularly Pk2, are crucial for proper NT formation in *Xenopus*.

### 2.2. The Pk2 C-terminus physically interacts with MLC9

**Figure 2.**
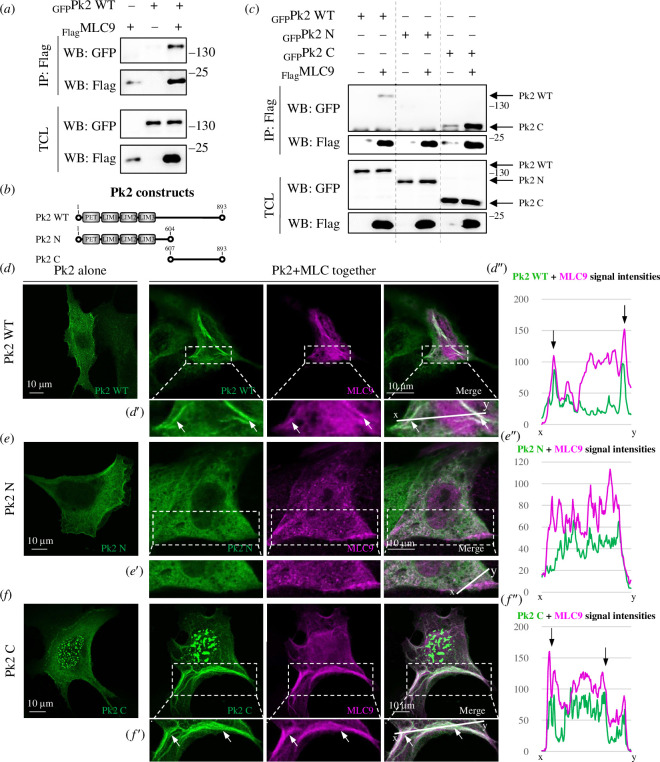
The C-terminus of Pk2 physically interacts with MLC9. (*a*) Co-IP of GFP-Pk2 and Flag-MLC9 in MDCK cells shows their physical binding in the MDCK cell lysate. (*b*) A scheme of the used Pk2 deletion constructs. (*c*) Co-IP of N- and C-terminal Pk2 constructs with MLC9 shows that the C-terminus of Pk2 is responsible for the interaction with MLC in the MDCK cell lysate. (*d–f*) IF of Pk2 constructs and MLC9 in MDCK cells shows colocalization of Pk2 WT and C-terminus with MLC in cells and their recruitment into fibre-like structures (white arrows). The scale bar is on the left bottom. (*d′–f*′) Regions of interest with the lines where the intensity profiles were measured. (*d″–f″*) Profiles of signal intensities show a colocalization of Pk2 WT and C-terminal with MLC9.

In addition to our initial studies in *Xenopus* embryos, we conducted biochemical analyses in MDCK (Madin-Darby canine kidney) cells, a well-established *in vitro* tissue culture model for dissecting actomyosin contractility [42]. To analyse the physical interaction between Pk2 and MLC9 in detail, we performed co-immunoprecipitation (co-IP) in lysates of MDCK cells with overexpressed Pk2 and MLC9. The pull-down analysis demonstrated the binding of Pk2 to MLC9 (figure 2*a*; electronic supplementary material, figure 3A).

**Figure 3.**
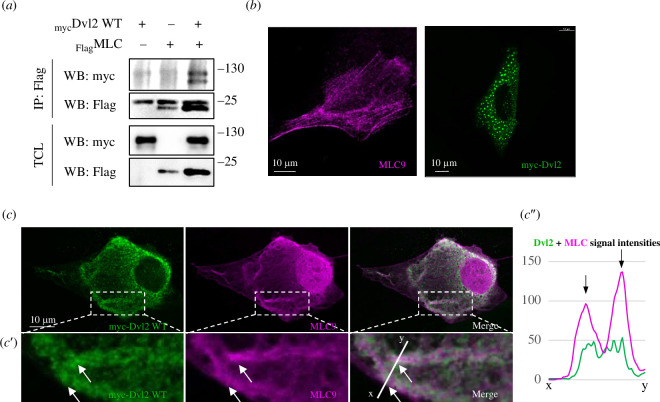
Dishevelled (Dvl) also physically interacts with MLC9. (*a*) Co-IP of myc-Dvl2 and Flag-MLC9 in MDCK cells shows their physical binding in the cell lysate. The upper signal in IP: Flag, WB: Flag is non-specific. (*b*) Subcellular localization of MLC9 and Dvl2 alone in MDCK cells. (*c*) IF of Dvl2 constructs and MLC in MDCK cells shows that Dvl2 interacts with MLC9 in cells and they both colocalize in fibre-like structures (white arrows). The scale bar is on the left bottom. (*c*′) Region of interest with a line where the intensity profile was measured. (*c*″) Profiles of signal intensities show the colocalization of Dvl2 and MLC9.

To determine which part of Pk2 mediates this interaction, we generated various deletion mutant constructs (figure 2*b*). Given that the Pk N-terminus is responsible for planar polarization and the Pk C-terminus for cell membrane binding, it was crucial to identify the relevant region. Our co-expression experiments with Pk2 terminus constructs revealed that only the Pk2 C-terminus, and not the Pk2 N-terminus, is responsible for the direct binding to MLC9 (figure 2*c*). To further explore this possibility, we co-transfected full-length Pk2 and Pk2 deletion mutants with MLC9 in MDCK cells and analysed their subcellular localization. MLC9 alone was localized into fibre-like structures in MDCK cells (figure 3*b*). Notably, when both Pk2 and MLC were co-expressed, MLC colocalized with Pk2 full-length and its C-terminus, but not the N-terminus, into these structures (figure 2*d–f* and white arrows in figure 2*d*′–*f′*).

To determine if these fibre-like structures are actin bundles, we repeated this experiment with actin staining and observed colocalization of both Pk2 WT and the C-terminus in actin bundles (electronic supplementary material, figure 2C–F). We measured intensity profiles of signals to analyse the colocalization (figure 2*d″*–*f*″; electronic supplementary material, figure 2C″−E″), with the measurement lines (figure 2*d′*–*f′*; electronic supplementary material, figure 2C′−E′), together showing the overlay of Pk2, MLC9 and actin signals.

We then asked whether it is possible to examine functionally the link between PCP proteins and actomyosin network in the tissue culture system. For that purpose, we investigated whether Pk2 can regulate cell volume, which potentially serves as an indicator of cytoskeletal activity in MDCK cells [43]. To assess this, we measured cell volumes using optical sectioning and analysed the samples with the Imaris software (electronic supplementary material, figure 2A). Our observations indicated that both Pk2 WT and the C-terminus led to a significant increase in cell volume, whereas cells expressing the Pk2 N-terminus did not exhibit such properties (electronic supplementary material, figure 2B). These findings suggest that Pk2 WT and its C-terminus may play a role in cytoskeletal activity in MDCK cells.

Based on these results, we concluded that the physical interaction between PCP and actomyosin on the level of Pk2 and MLC9 occurs via the Pk2 C-terminus.

### 2.3. Dvl2 and Dvl3 also physically interact with MLC9, and both Pk2 and Dvl2 bind to MLC9 independently of their transmembrane PCP partners

After confirming that Pk2 can bind to MLC9, we sought to test whether Dvl, another cytoplasmic protein located at the opposite end of the PCP complex from Pk2, also interacts with MLC. Indeed, we detected the binding of Dvl2 and MLC9 in immunoprecipitated MDCK cell lysates (figure 3*a*; electronic supplementary material, figure 3B). To examine whether MLC9 colocalizes with Dvl2, we analysed their subcellular localization in MDCK cells and observed some overlap in fibre-like structures (figure 3*c*), though not as pronounced as with Pk2 (compared with figure 2*d–f*). A line drawn through the region of interest (figure 3*c*) allowed for intensity profile measurement, revealing colocalization of the two proteins (figure 3*c″*). Interestingly, the presence of MLC9 significantly influenced the localization of Dvl2, which typically forms dots or puncta (figure 3*b*). In addition, we wanted to test whether another isoform such as Dvl3 can bind to MLC. Indeed, we observed the interaction as in the case with Dvl2 (electronic supplementary material, figure 3B). These results confirmed physical binding between Dvl isoforms and MLC9.

We then explored whether the interaction of PCP proteins with MLC depends on their transmembrane protein partners, Vangl and Fzd, to gain insights into the underlying mechanism. To determine if Pk2 can interact with MLC in the absence of its binding partner Vangl, which anchors Pk2 to the cell membrane (e.g. [16,18,20,22,44–47]), we performed a co-IP of Pk2 and MLC9 in Vangl1-2 knockout (KO) HEK293 cells [48]. Western blot (WB) results showed that Pk2 and MLC9 interact even without Vangl1−2 (electronic supplementary material, figure 3C). Similarly, we conducted a co-IP experiment with Dvl2 and MLC9 in Fzd1−10 KO HEK293 cells [49]. The results indicated that the binding between Dvl2 and MLC is independent of its binding partners Fzd1–10 (figure 3*c′*).

Collectively, these findings demonstrate that Dvl2 can also bind to MLC9 and that the interactions of both Pk2 and Dvl2 with MLC9 are independent of their transmembrane PCP protein partners. This supports our hypothesis that cytoplasmic PCP proteins can be readily recruited to the actomyosin network.

### 2.4. Cytoplasmic PCP proteins increase levels of pMLC

Actomyosin contractility depends on MLC’s phosphorylation and its enrichment at the apical cell membrane [3], processes induced by the Rho-associated kinase (ROCK) among others (electronic supplementary material, figure 4A) [50,51,4]. To assess whether Pk2 can increase the level of phosphorylated MLC (pMLC), we analysed the phosphorylation of exogenous MLC9 in MDCK cells by WB and examined the phosphorylation distribution of endogenous MLC in the *Xenopus* neural plate by immunostaining (electronic supplementary material, figure 4A, *bottom*).

**Figure 4.**
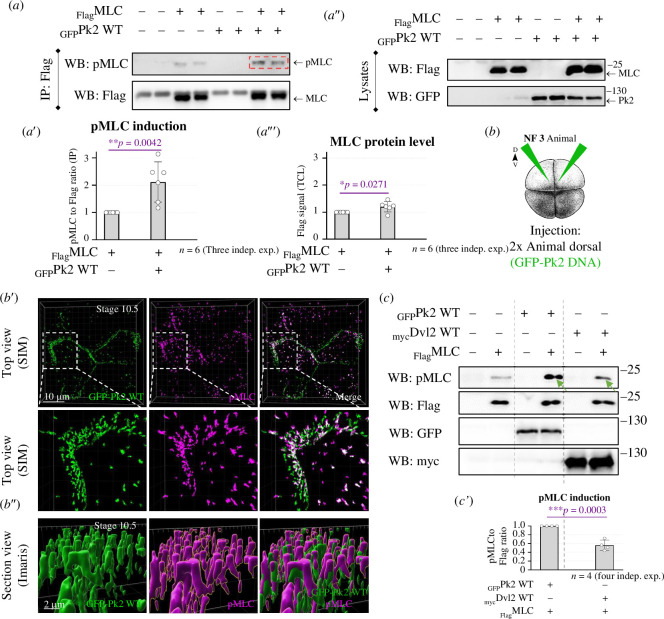
Pk2 increases the levels of both exogenous and endogenous pMLC. (*a–a′″*) The increase of exogenous pMLC by Pk2 (red dashed box) in MDCK cells detected by WB approach. In (*a′*), the graph with three independent experiments and the *t*‐test is shown. **p ≤ 0.01. In (*a′,a′″*), cell lysates of (*a*) are shown; *p ≤ 0.05. (*b*) *Xenopus* embryos were injected with Pk2 DNA into both animal dorsal blastomeres at the four-cell stage embryos. (*b*′) Pk2 induces endogenous pMLC in *Xenopus* ectoderm, NF stage 10.5. The scale bar is on left bottom. (*b″*) Data from (*b)* were processed and visualized in Imaris and show that pMLC is on top of Pk2 protein clusters, suggesting their apical (co)localization. The scale bar is on left bottom. (*c–c′*) Not only Pk2, but also Dvl2 induces exogenous pMLC in MDCK cells (green arrows). The Dvl2 efficiency in pMLC was about 50% of Pk2; ***p ≤ 0.001.

WB analysis of immunoprecipitated MDCK cell lysates showed an enrichment of pMLC signal for exogenous MLC9 after Pk2 co-expression, indicating that Pk2 increased the level of pMLC (figure 4*a,a′*). Analysis of cell lysates confirmed that the increased pMLC signal was not due to altered protein expression, which was only slightly increased in conditions with both Pk2 and MLC9 (figure 4*a″,a′″*). Nevertheless, these changes were already accounted for in the final pMLC/Flag ratios and were negligible in contrast to pMLC induction.

Then, we investigated whether a similar effect occurred at the endogenous MLC level. We injected Pk2 DNA into both dorsal blastomere of the four-cell stage *Xenopus* embryos to achieve mosaic expression (figure 4*b*) and, using immunostaining and SIM (structured illumination microscopy), observed an enrichment of pMLC signal in the *Xenopus* ectoderm in regions with prominent Pk2 expression (figure 4*b*). Given the importance of pMLC localization for actomyosin contractility, we further analysed the subcellular localization of pMLC. Closer inspection revealed using the Imaris software that the Pk2-induced pMLC signal was apically localized in our experimental system (figure 4*b″*). Collectively, these results suggest that Pk2 can induce pMLC both *in vitro* and *in vivo*, with the pMLC signal localized on the apical cell surface.

After confirming the physical interaction with MLC9, we examined whether Dvl, like Pk2, can also promote phosphorylation of MLC. Indeed, Dvl2 was able to induce pMLC (figure 4*c*), albeit to a lesser extent compared with Pk2. We observed that Dvl2 induced increased levels of pMLC at approximately 50% of the level induced by Pk2 in our *in vitro* experimental system (figure 4*c′*). Nevertheless, both PCP proteins can induce pMLC, indicating that Pk2 and Dvl2 contribute to the regulation of actomyosin contractility.

### 2.5. Both PCP proteins seem to act predominantly through Rap1 small GTPase and CK1ε kinase

The previous findings prompted a detailed investigation into the molecular mechanism driving pMLC induction by PCP proteins. Given the established role of small GTPases in this process [4,50,51], we initially examined the major players, RhoA and Rac1. Surprisingly, active levels of neither RhoA nor Rac1 showed an increase following Pk2 and Dvl2 overexpression (electronic supplementary material, figure 4B). However, inhibition of ROCK kinase and Rac1 resulted in a decreased pMLC signal, suggesting an indirect influence of these GTPases (electronic supplementary material, figure 4C). These observations imply that while Rho and Rac1 are likely involved in PCP-dependent pMLC events, their role may be indirect.

**Figure 5.**
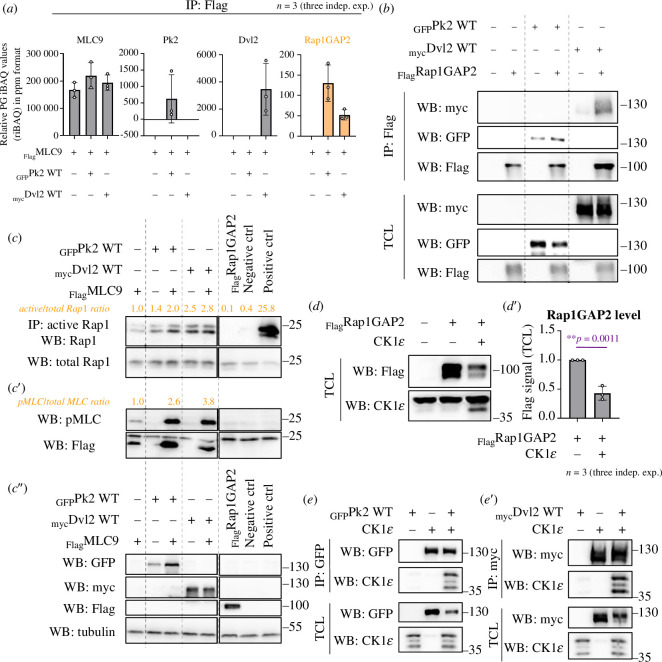
Both PCP proteins appear to function through the Rap1 small GTPases and CK1ε pathway. (*a*) Mass spectrometry analysis identifies candidates for MLC phosphorylation induced by Pk2 and Dvl2, with Rap1 GTPase activating protein (Rap1GAP2) emerging as a key candidate that interacts with MLC only in the presence of Pk2 or Dvl2. (*b*) Co-IP of Pk2, Dvl2 and Rap1GAP2 in MDCK cells demonstrates a physical interaction between PCP proteins and Rap1GAP2. (*c*) Both Pk2 and Dvl2 increase the total level of Rap1 small GTPase in MDCK cell lysates, suggesting its involvement in this process, as shown through Rap1 activation assay. The Rap1 inhibitor GGTI298 and Rap1GAP2 were used as negative controls for WB signal detection. (*d–d*′) Casein kinase 1ε (CK1ε) decreases the protein level of Rap1GAP2 in MDCK cells. In (*d*′), the graph shows results from three independent experiments with statistical significance indicated by a *t*‐test (**p ≤ 0.01). (*e–e*′) Co-IP of Pk2 and Dvl2 with CK1ε reveals a physical interaction between both PCP proteins and CK1ε in MDCK cells.

To elucidate potential binding partners responsible for MLC phosphorylation, we conducted mass spectrometry (MS) analysis on co-IPed samples of MDCK cell lysates with Pk2, Dvl2 and MLC9 overexpression. Among the identified targets, Rap1 GTPase activating protein 2 (Rap1GAP2), which is an inhibitor of Rap1 small GTPase [52], emerged (figure 5*a*). Furthermore, co-IP experiments confirmed a physical interaction between both PCP proteins and Rap1GAP2 (figure 5*b*).

The activation status of Rap1 was found to be crucial for MLC phosphorylation by PCP proteins. To assess Rap1 activity, a Rap1 assay was conducted, revealing an increase in active Rap1 upon co-expression with Pk2 or Dvl2 (figure 5*c*). These findings suggest that MLC phosphorylation by PCP proteins occurs via the Rap1 small GTPase pathway.

Given Rap1GAP2’s inhibitory effect on Rap1, we sought a potential inhibitor of Rap1GAP2 to ensure Rap1 activation for MLC phosphorylation. The co-expression of Pk2 and Dvl2 did not change the protein level of Rap1GAP2 (see figure 5*b*). Based on Tsai *et al*.’s data [53] on CK1ε and SIPA1L1, another Rap1GAP, which demonstrated a positive CK1 involvement in Rap1 signalling during convergent extension in vertebrates, we speculated that this kinase linked to PCP [54] might also be active in our case with Rap1GAP2 and neurulation. Indeed, co-expression of CK1ε with Rap1GAP2 in MDCK cells resulted in a significant decrease in Rap1GAP2 protein levels (figure 5*d–d′*), indicating potential inhibition of this Rap1 inhibitor by CK1ε. Additionally, to confirm that CK1ε binds to Dvl and Pk in our experimental setup to prove its relevance to PCP and polarization, we performed co-IP assays. The results revealed physical interactions between Pk2, Dvl2 and CK1ε (figure 5*e–e′*), suggesting that PCP proteins can facilitate the spatiotemporal recruitment of CK1ε to locally degrade Rap1GAP2. This, in turn, activates Rap1 and leads to MLC phosphorylation (see below).

### 2.6. Both Pk2 and Dvl2 locally induce endogenous pMLC in the *Xenopus* neural plate and their overexpression causes neural tube defects

**Figure 6.**
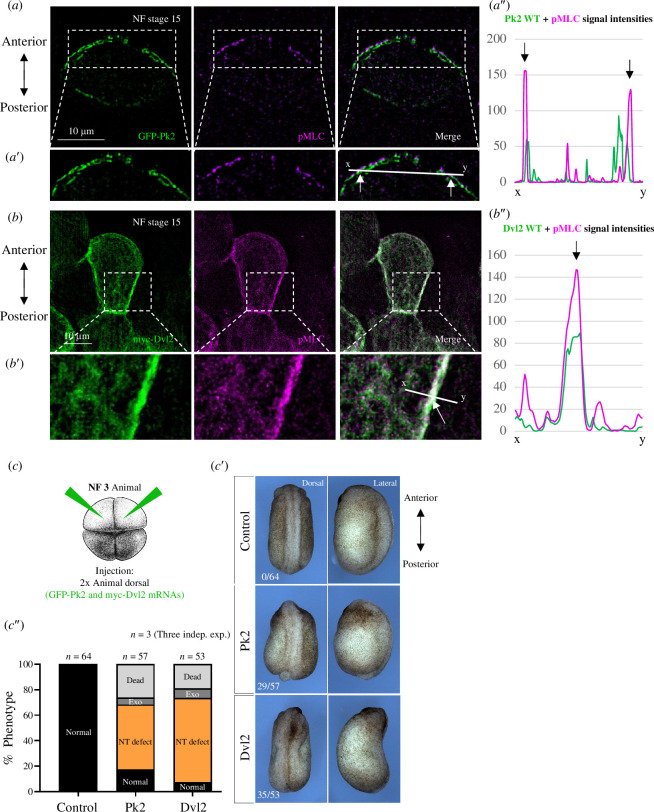
Both Pk2 and Dvl2 locally induce endogenous pMLC in the *Xenopus* neural plate and their overexpression causes NT defects. (*a–a″*). Anteriorly localized Pk2 locally induces pMLC (white arrows) in the *Xenopus* neural plate, NF stage 15. In (*a*″), profiles of signal intensities show colocalization of Pk2 and pMLC. (*b–b″*) Posteriorly localized Dvl2 also colocalizes with induced pMLC in the *Xenopus* neural plate, NF stage 15. In (*b″*), profiles of signal intensities show colocalization of Pk2 and pMLC. (*c–c″*) Overexpression of Pk2 and Dvl2 in *Xenopus* embryos. In (*c)*, embryos were injected in four-cell stage into two animal dorsal blastomeres with Pk2 or Dvl2 mRNA. (*c′*) Phenotype of Pk2 and Dvl2 overexpression shows mostly NT defects. The numbers in the bottom right corner correspond to the amount of embryos with NT defects versus the total amount. (*c″*) A graph of different phenotypes. Most of the embryos with overexpression of Pk2 and Dvl2 suffered from NT defects, highlighting the key role of both proteins during vertebrate neurulation.

To gain further insight into the capacity of Pk2 and Dvl2 to induce pMLC and its relevance *in vivo*, we utilized the neural plate of *Xenopus* embryos as a model system. Specifically, we injected Pk2 and Dvl2 plasmid DNAs into both dorsal blastomere of the four-cell stage *Xenopus* embryos to achieve mosaic expression. Following embryo fixation, the stained neural plates were visualized using SIM. We observed anteriorly localized and membrane-bound Pk2 with a clearly induced endogenous pMLC (figure 6*a*), corroborating previous *in vitro* findings in MDCK cells. Although Dvl2 was not consistently posteriorly polarized in all cases (electronic supplementary material, figure 5), it still induced endogenous pMLC at locations overlapping with Dvl expression on the cell membrane (figure 6*b*). The lack of consistent polarization does not negate Dvl2’s ability to induce pMLC (electronic supplementary material, figure 5, *right*).

To confirm the colocalization of Pk2 and induced pMLC, we measured intensity profiles along a defined line (figure 6*a′*). The intensity profiles confirmed colocalization of both signals (figure 6*a″*). A similar result was obtained from the intensity profiles of Dvl2 and induced pMLC (figure 6*b′*–*b″*), thus suggesting both overexpressed PCP proteins are involved in increased pMLC signal *in vivo*.

To assess the phenotypic effects of Pk2 and Dvl2 overexpression and its impact on NT closure, we injected mRNA coding for these proteins into two dorsal blastomeres of the four-cell stage embryos. Most of the embryos injected with either Pk2 or Dvl2 exhibited NT defects (figure 6*c–c″*), suggesting that the NT defects caused by the overexpression of PCP proteins happen due to the overactivation of pMLC.

To further validate our observation, we employed live imaging techniques to detect changes at NF stage 10.5. Utilizing an experimental setup similar to that shown in figure 4*b* with mosaic expression, we observed the dynamic behaviour of Pk2-expressing cells. As demonstrated in electronic supplementary material, video S1, these cells underwent noticeable shrinking, a process closely resembling apical constriction. These live imaging data provide evidence supporting our hypothesis, showcasing the real-time cellular dynamics associated with Pk2 expression and its ‘novel’ role in apical constriction and actomyosin contractility.

**Figure 7.**
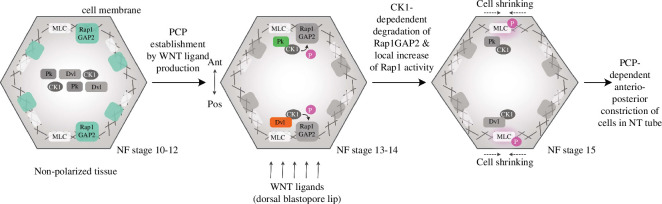
A suggested mechanism of PCP-dependent activation of cell contractility through the binding of cytoplasmic PCP proteins prickle (Pk), dishevelled (Dvl) and casein kinase 1ε (CK1) to actomyosin proteins Rap1GAP2 and myosin light chain (MLC), leading to its phosphorylation (pMLC) through Rap1. This model is based on our experimental data in MDCK cells and in the neural plate of *Xenopus* embryos. We suggest that the key event in this process is the right balance of pMLC levels, which are regulated by cytoplasmic PCP proteins. Data about the dorsal blastopore lip, WNT ligand secretion and PCP establishment are demonstrated elsewhere (see the accompanying text).

In summary, these results provide solid evidence for the capacity of PCP proteins to phosphorylate MLC under physiological conditions in a well-established *in vivo* model. Based on the findings presented in this study, we propose a new model of MLC phosphorylation mediated by different members of the PCP pathway. We suggest that the activation of actomyosin contractility occurs through the direct binding of PCP proteins—Pk on the anterior side, Dvl on the posterior side and CK1ε on both sides—to actomyosin proteins Rap1GAP2 and MLC, leading to subsequent phosphorylation via Rap1. This indicates that MLC phosphorylation and its proper balance is an important requirement during NT closure in vertebrates (figure 7).

## 3. Discussion

Understanding the intricate organization of the cytoskeletal architecture in response to external signals such as cellular signalling is a fundamental challenge in the fields of cell and developmental biology, as it underpins the development of specific tissue and cellular morphologies. In this study, we aimed to investigate a novel connection between signalling pathways mediated by PCP and the actomyosin contractility network. To address this, we focused on the two main cytoplasmic PCP proteins, Pk2 and Dvl2 and explored their potential interaction with MLC9 and the Rap1-dependent protein network. Our findings suggest a relationship between PCP protein and actomyosin contractility, mediated by Rap1-dependent MLC phosphorylation (pMLC) via CK1ε-dependent degradation of Rap1GAP2 (figure 7). This proposed link between PCP signalling and actomyosin contractility in NT closure prompts several key points for discussion.

The first point concerns the ‘novel’ role of Pk in myosin activity. While Dvl is widely implicated in the mechanism by which PCP proteins influence myosin activity [26,28], there is a question of whether it is the only PCP ‘player’ involved in actomyosin contractility. This importance of Dvl might have arisen from pioneering work in *Drosophila*, where cytoskeletal rearrangements like actin hair (or trichome) growth are engaged on the side of Dvl [16–22]. Earlier work has also shown that the PCP pathway exhibits an asymmetric protein localization along this pathway, which is a crucial characteristic for its function (e.g. [22]). Thus, Dvl and Pk are localized at opposite poles of the cell (see figure 1*a*), and there is a strong theoretical assumption that both sides should then be involved in actomyosin contractility, especially during symmetrical events such as NT closure. This was where we started considering Pk as another potential linker for PCP–actomyosin crosstalk as well. Therefore, we primarily focused here on studying the role of Pk2 in actomyosin contractility. While Dvl has been shown to be indirectly involved in actomyosin contractility, e.g. during neurulation [28], there is limited evidence regarding the role of Pk in these events. In fact, the article by Newman-Smith *et al*. [32] is the first to demonstrate that myosin is anteriorly polarized in the invertebrate Ciona notochord. The authors found that actomyosin cytoskeleton disruption led to a loss of Pk and its binding PCP partner Vangl’s polarization in the Ciona notochord. Their results indicate that myosin is necessary for PCP protein localization and vice versa, and they highlight a relationship between the actomyosin cytoskeleton and anterior PCP components [32]. Moreover, approximately one-third of the cells in the Pk mutant line exhibit altered myosin. This suggests a connection between anterior PCP signalling and the localization of myosin, implying that they should be somehow linked [32]. Furthermore, there is additional evidence supporting the connection between Pk and actomyosin contractility from *Xenopus* [31]. The authors found a correlation between increased PCP protein presence and the behaviour of actomyosin contraction. This suggests an interplay between the localization of PCP proteins, including Pk, and the actomyosin network in the closing vertebrate NT. Moreover, the partial MLC9 and Pk2 colocalization in the *Xenopus* neural plate shown in that work [31] supports our results that confirm the physical interaction between Pk2 and MLC9 (figure 2). Our observation that Pk2 is involved in actomyosin contractility is consistent with a previous report [55], which states that Vangl2, the Pk binding partner, is required for the formation of the NT as a result of complex morphogenetic movements in vertebrates. Continuing along the same line, another study discusses the role of Vangl2 in actomyosin contractility, and the authors demonstrate that there is a decrease in pMLC (see the third paragraph of §3), specifically at the blastopore closure in frog embryos with depleted Vangl2 [56]. In sum, these findings together with our results provide solid support for the suggested relationship between Pk from the anterior PCP complex and actomyosin contractility.

The second point is about Dvl, which has been shown in various model systems to interact with actomyosin contractility, not only in *Drosophila* but also in vertebrates [26,28]. Previous studies have notably linked Shroom3, a major regulator of apical constriction [11], as a critical mediator between PCP through Dvl and actomyosin contractility, particularly in vertebrate NT morphogenesis. The work by McGreevy *et al*. [27] provides compelling evidence for the involvement of the Dvl2–Shroom3–ROCK pathway in linking planar polarization to directional contractility, essential for NT closure in mice. Additionally, emerging evidence suggests the existence of extended signalling complexes called WERDS, comprising Wnt ligands, ephrinB2, Ror2 receptor, Dvl and Shroom3 [57]. Furthermore, Dvl has been shown to interact during various events with RhoGEFs (guanine nucleotide exchange factors) such as PDZ-RhoGEF [28], WGEF, p114-RhoGEF [58], Trio [59] and Lfc [58] (also known as GEF-H1). However, it remains unclear which of the aforementioned GEFs (and to what extent) are involved in PCP signalling [12]. Moreover, Dvl2 could also be linked to actomyosin contractility through protein tyrosine kinase 7 (PTK7) [42,60]. Andreeva *et al*. [42] revealed another aspect of actomyosin regulation in intercellular junctions, providing evidence for the involvement of PTK7-Src signalling in the regulation of ROCK activity and spatial organization of the actomyosin network, which led to junctional contractility regulation, and changes in cell shape and PCP signalling. Thus, PTK7 plays a role in processes regulated by PCP signalling [60]. Taking all this evidence into consideration, however, we describe and suggest here a mechanism by proposing a ‘simple’ relationship between PCP proteins such as Dvl2 and the actomyosin network represented by MLC9, together with proteins such as CK1ɛ and Rap1 (see figure 7). Last but not least, this mechanism is likely applicable to both cell movements such as intercalation and cell shape changes such as apical constriction, all mediated by actomyosin contractile apparatus [10–12] at the same time or place and which are hard to dissect and study in an experimental model.

As a third point, we briefly discuss the physiological readout of the proposed interaction between cytoplasmic PCP proteins and MLC, which may be the activation of myosin II by pMLC in Ser19. Such a pMLC event serves as the general readout for actomyosin contractility and represents the downstream effect of our results, which is also supported by the findings reported by the Wallingford group [37], where the authors observed pMLC enrichment coinciding with PCP protein localization during convergent extension, i.e. on the anterior and posterior cell edges. The pattern of contraction based on pMLC described in this article [37] is consistent with reports of PCP proteins’ localization on the anteroposterior cell faces in the *Xenopus* [28], chick [61] and mouse [62] NT. Furthermore, the group of Wallingford also demonstrated that disrupting Dvl in the *Xenopus* gastrula mesoderm led to a reduction in overall pMLC levels [37], which further supports our results with the interaction between PCP proteins and MLC. These data were further supported by studies conducted not only in *Drosophila* [30], but also in *Xenopus*, chickens and mice [27,28,31]. All these evidences support the results presented in our work regarding the interaction between PCP signalling and actomyosin contractility reflected by increased levels of pMLC (see figures 4 and 6).

Finally, we present the novel interaction network involving PCP signalling and Rap1 GTPase (figure 5). Our data strongly support the hypothesis that these interactions lead to the activation of MLC through pMLC induction by Rap1, facilitated by Rap1GAP2 degradation mediated by CK1ɛ activity. We speculate that CK1ɛ is brought to Rap1GAP2 by Pk and Dvl, which are polarized during vertebrate neurulation. A similar result with Rap1, CK1ɛ and non-canonical Wnt signalling has been demonstrated in the case of SIPA1L1, another Rap GTPase activating protein and convergent extension in vertebrates [53]. These findings further support our results with neurulation and PCP proteins.

In conclusion, we identified a novel interaction network involving Pk2, Dvl2, CK1ɛ, Rap1GAP2 and Rap1 GTPase, which leads to MLC activation through pMLC induction by Rap1, facilitated by CK1ɛ-mediated phosphorylation of Rap1GAP2, which leads to its degradation. This mechanism links PCP signalling to the actomyosin contractility network during vertebrate neurulation (figure 7) and implements in general the work shown elsewhere, e.g. Wnt ligand secretion by the dorsal blastopore lip and the spatiotemporal alignment of PCP proteins in the neural plate [12,63].

## 4. Material and methods

### 4.1. Cell culture maintenance

The MDCK and HEK293 cells were kept in an incubator at 37°C with 5% CO_2_. Both cell lines and their derivates were grown in Dulbecco’s modified Eagle medium (DMEM; cat. no. REF41966-029, Gibco), supplemented with 10% (v/v) foetal bovine serum (FBS; lot no. 41Q9221K, cat. no. 10270, Gibco), 1% (v/v) penicillin–streptomycin (cat. no. SV30010, Hyclone, Biotech) and 1% l-glutamine (cat. no. 25030024, Life Technologies).

As for co-IP, cells were seeded on 6 cm plates with 15% confluency in the case of MDCK cells and 30% confluency in the case of HEK293 cells and transfected with 2 µg of plasmid DNA (jetOPTIMUS kit, 101000051, Polyplus) per plate for 24 h. As for IF, cells were seeded with 10% confluency on 24-well plates with gelatin-coated coverslips and transfected with 0.4 µg of plasmid DNA (jetOPTIMUS kit, 101000051, Polyplus) per well for 24 h. For Rho, Rac1 and Rap1 assays, cells were seeded on 10 cm plates with 15% confluency and transfected with 6 µg of plasmid DNA per plate for 24 h. As for MS, cells were seeded on 10 cm plates (each condition consisted of two plates) with 15% confluency and transfected with 6 µg of plasmid DNA (jetOPTIMUS kit, 101000051, Polyplus) per plate for 24 h.

For transfection, the following plasmids were used: GFP-xPk2 aa 1-893 [31], of which both GFP-xPk2 1-604 (Pk2 N) and GFP-xPrickle2 607-893 (Pk2 C) were derived by site-directed mutagenesis according to the instructions of the manufacturer (QuikChange II XL, 200522, Agilent; for primers, see electronic supplementary material, table S1) and subsequently confirmed by sequencing; then myc-xDvl2 [64], hRap1GAP2-Flag [52], HA-hDVL3 [65], hCK1ε [65], constitutively active RhoA-V14 and Rac1-V12 [66] and Flag-cMLC9, which has been subcloned from GFP-cMLC9 [67] into the empty and linearized pCS2-Flag (N-terminally tagged) vector through XhoI and XbaI restriction sites (for primers, see electronic supplementary material, table S2). Control DNA plasmid pcDNA3.1 [65] was used to optimize the DNA transfection amount (note: h, human; m, mouse; x, *Xenopus*; c, chicken isoforms).

In the case of inhibitors, ROCK inhibitor Y-27632 (final concentration 10 µM in DMSO, cat. no. SCM075, Sigma Aldrich) and Rac1 inhibitor NSC23766 (10 µM in DMSO, cat. no. HY-15723A, MedChemExpress) were added into the medium the day after transfection and kept there until cell lysis. Rap1 inhibitor GGTI298 trifluoroacetate (5 µM in DMSO, cat. no. HY-15871, MedChemExpress) was added 4 h after transfection for overnight incubation and in the case of 3 h incubation, the inhibitor was added the next day after transfection and kept there until cell lysis.

### 4.2. Co-immunoprecipitation

First, once the medium had been removed from the 6 cm plates, 1 ml of PBS was added to wash each plate (MDCK cells were washed twice). The following lysis buffer was prepared with the following concentrations in order to obtain a cell lysate: 50 mM Tris (pH 7.5), 150 mM NaCl, 1 mM EDTA, 0.5% of NP40, with the addition of 1× protease inhibitor (11836145001, Roche), 1 nM DL-dithiothreitol (DTT) and 1× phosphatase inhibitor cocktail II (524625, Merck). Then, 1 ml of the lysis buffer was added to each plate and left for 20 min on ice at 4°C. The obtained cell lysates were centrifuged at 14 400 rcf at 4°C for 15 minutes. Next, two types of samples were separated from the supernatant obtained in each plate; 60 µl was kept as the total cell lysate (TCL), and 800 µl was used for the co-immunoprecipitation (co-IP). To the TCL samples, 20 µl of 4× Laemmli buffer was added and stored at −20°C until the SDS-polyacrylamide gel electrophoresis (SDS-PAGE) was performed. In the case of most experiments, 1 µg of the primary antibody (Flag M2, F1804, Sigma, or GFP, Fitzgerald, 20R-GR-011, or myc, C3956, Sigma) was added and left to incubate on ice for 1 h to carry out the antigen–antibody reaction. Finally, 10 µl of the G-protein sepharose beads (Protein G sepharose 4 fast flow, 17-0618-01, GE Healthcare), previously washed with lysis buffer (centrifuged at 0.1 rcf at 4°C for 1 min), were added and left to incubate overnight under agitation. In the case of some co-IPs with Flag tag, 10 µl of Flag M2 beads (Flag M2 Affinity Gel, A2220-1ML, Millipore/Merck) were added into samples for immunoprecipitation and left to incubate for 3 h under agitation at 4°C. The beads were previously washed with lysis buffer (centrifuged at 0.1 rcf at 4°C for 1 min). After incubation, the samples were washed five times with 800 µl of lysis buffer without protease and phosphatase inhibitors and centrifuged for 1 min, 0.1 rcf at 4°C. After removing the supernatant, 33 µl of 2× standard Laemmli buffer was added to each sample. Both IP and TCL samples were boiled (10 min at 70°C) for analysis by SDS-PAGE and continued with WB.

### 4.3. Rho, Rac1 and Rap1 assays

Samples for Rho, Rac1 and Rap1 assays were prepared according to the manufacturer’s protocols from the kits (Active Rho—Pull Down and Detection Kit, 16116, ThermoFisher; and Active Rac1—Pull Down and Detection Kit, 16118, ThermoFisher; Rap1 Activation Assay Kit, 17-321, all Sigma-Aldrich) and continued with WB.

### 4.4. Western blot

WB membranes were developed using the fusion imaging system (Amersham ECL Prime Luminol Enhancer Solution, 29018903, Cytiva) chemiluminescence documentation system. The primary antibodies used were: pMLC pS20 (equivalent to Ser19) (ab2480, Abcam—dilution 1:1000); myc (C 3956 Sigma—1:1000); GFP (20R-GR-011, Fitzgerald—1:1000), Flag (7425, Sigma-Aldrich—1:1000); GFP B-2 (sc-9996, Santa Cruz—1:500); Flag M2 (F1804, Sigma/Merck—1:1000); c-myc 9E10 (sc-40, SC Exbio—1:500); CK1ε (610446, BD Transduction Laboratories—1:750); HA 11 (MMS-101R, Covance—1:1000); and Tubulin (t6793, Merck—1:1000). The antibodies against small GTPases were a part of particular kits from Sigma.

The corresponding secondary antibodies were mouse-HRP (A4416, Sigma—dilution 1:5000) and rabbit-HRP (A0545, Sigma—1:5000).

### 4.5. Mass spectrometry

For mass spectrometry (MS), the samples were prepared by using co-IP. The procedure was the same as described in the co-IP section, but some quantities differed. MDCK cells were seeded on 10 cm plates, two plates for each condition. Each plate was washed with 8 ml of standard PBS buffer and 500 µl of lysis buffer was added. For IP, 1 ml of supernatant was used, followed by the addition of 15 µl of Flag M2 beads (Flag M2 Affinity Gel, A2220-1ML, Millipore/Merck) into samples for IP. After incubation, the samples were washed three times with 1 ml of lysis buffer without protease and phosphatase inhibitors and three times with 1 ml of the lysis buffer without inhibitors and NP40. After removing the supernatant, no Laemmli buffer was added, and the samples were stored at 4°C before the MS procedure.

Bead-bound protein complexes were then digested directly on beads according to Hollenstein *et al*. [68] using 0.15 μg of LysC (MS grade, Promega) for overnight digestion (25°C, dark), then only supernatant was further digested with 0.75 μg of trypsin (Proteomics grade; Merck) for 5 h at 37°C. The peptide mixture was evaporated completely in a SpeedVac concentrator (Thermo Fisher Scientific) and extracted into LC-MS vials by 2.5% formic acid (FA) in 50% acetonitrile (ACN) and 100% ACN with the addition of polyethylene glycol (20 000; final concentration 0.001%) [69] and concentrated again in a SpeedVac concentrator (Thermo Fisher Scientific) to 15 μl to eliminate the ACN prior the LC-MS analyses.

LC-MS/MS analyses of all peptides were done using an UltiMate 3000 RSLCnano system (Thermo Fisher Scientific) connected to an Orbitrap Exploris 480 spectrometer (Thermo Fisher Scientific). Prior to LC separation, tryptic digests were online concentrated and desalted using a trapping column (C18 PepMap Neo, dimensions 300 μm ID, 5 mm long, 5 μm particles, Thermo Fisher Scientific). After washing of trapping column with 0.1% TFA, the peptides were eluted in backflush mode (flow 300 nl min^–1^) from the trapping column onto an analytical separation column (Ion Opticks, Aurora C18, third generation, 25 cm long, 75 μm inner diameter, 1.7 μm particles) by 90 min gradient programme (flow rate 150 nl min^−1^, 3–42% of mobile phase B; mobile phase A: 0.1% FA in water; mobile phase B: 0.1% FA in 80% ACN). Equilibration of the trapping column and the analytical column was done prior to sample injection into the sample loop. The analytical column was installed in an EASY-Spray ion source (Thermo Fisher Scientific) according to the manufacturer’s instructions with a column temperature of 50°C.

MS data were acquired in a data-dependent strategy (cycle time of 2 s). The survey scan range was set to *m*/*z* 350–2000 with a resolution of 120 000 (at *m*/*z* 200), normalized target value of 250% and a maximum injection time of 500 ms. HCD MS/MS spectra (isolation window *m*/*z* 1.2, 30% relative fragmentation energy) were acquired from *m*/*z* 120 with a relative target value of 50% (intensity threshold 5 × 103), resolution of 15 000 (at *m*/*z* 200) and maximum injection time of 50 ms. Dynamic exclusion was enabled for 45 s.

### 4.6. Immunofluorescence of tissue culture cells

The next day after transfection, cells were fixed by adding 250 µl of a solution containing 4% of paraformaldehyde and blocked in PBTA (3% (w/v) BSA, 0.25% Triton) for 1  h and incubated overnight with primary antibodies (GFP mouse, Millipore—1:500; Flag rabbit, Sigma—1:500 in the case of Pk2 and MLC9, or r-myc rabbit, C 3956, Sigma—1:500; Flag mouse M2—1:500 in the case of Dvl2 and MLC9) in PBTA at 4°C. The next day after washing with PBS, the secondary antibodies (Alexa Fluor Mouse 488, Alexa, A28175—1:500; Alexa Fluor Rabbit 568, Alexa, ab175471—1:500, and Alexa Fluor mouse 680, A10043—1:500 in the case of Pk2 and MLC; or Alexa Fluor Rabbit 488, Alexa—1:500; Alexa Fluor Mouse 568, Alexa—1:500 in the case of Dvl2 and MLC, Cy3 AffiniPure Donkey Anti-Rabbit IgG (H+L), 711-165-152, Jackson ImmunoResearch—1:500 for myosin II staining and Alexa Fluor Phalloidin 568 A12380—1:1000 for actin staining) were incubated in PBTA, washed with PBS and stained with DAPI (1:1000). All coverslips were mounted on microscopic slides. Cells were visualized using confocal microscopy (see §4.8).

### 4.7. *Xenopus* embryos

The work with *Xenopus laevis* was carried out according to the Czech animal use and care law and approved by local authorities and committees (MSMT-30784/2022-1; Animal Care and Housing Approval: 45055/2020-MZE-18134, Ministry of Agriculture of the Czech Republic). *Xenopus* embryos’ generation and cultivation were performed following standard protocols. Briefly, testes from males under anaesthesia (20% MS-222, Sigma-Aldrich, A5040) were removed surgically from the body cavity and transferred to cold 1× Marc’s modified ringers (MMR; 100 mM NaCl, 2 mM KCl, 1 mM MgSO_4_, 2 mM CaCl_2_, 5 mM HEPES, buffered to pH 7.4) supplemented with 50 µg ml^−1^ of gentamycin (Sigma-Aldrich, G3632). To induce egg laying, fully mature *Xenopus* females were injected with 260 U of human chorionic gonadotropin (hCG; Merck, Ovitrelle 250G) into the dorsal lymph sac for about 12–16 h before use and kept overnight at 18°C. Then, eggs were squeezed from an induced female directly into a Petri dish and mixed with a piece of testes in 0.1× MMR. After *in vitro* fertilization, embryos were cultivated in 0.1× MMR at 18–21°C. Embryos were staged according to the standardized table of Zahn *et al*. [70].

In figures 1*b*, 4*b*′ and 6*a*,*b* the embryos were microinjected with 100 pg of plasmid DNA in the four-cell stage into two dorsal blastomeres to express a protein of interest in a mosaic manner. After the injections, the embryos were left to develop to NF stage 10 to observe the ectoderm or to NF stage 15 to analyse the polarized neuroectoderm and then were fixed in 2% TCA (T0699, Sigma Aldrich) for 1 h.

All embryos were microinjected in a solution of 0.1× MMR with 2% Ficoll. The mRNAs were synthesized using mMessage mMachine kits (Ambion) and injected into two dorsal blastomeres of a four-cell stage or eight-cell stage embryo at the following concentrations: 200 pg of Pk2-GFP and 200 pg of Dvl2-GFP (figure 6*c*). For morpholino injections (figure 1*c*), 4 ng was injected into one cell at the eight-cell stage. The splicing Pk2-MO (GAACCCAAACAAACACTTACCTGTT) is complementary to the region spanning the 3′ end of exon 3 and 5′ end of intron 3, designed by this study [71]. Antisense morpholino for Dvl2 (5′-GGTAAATCACTTTAGTCTCCGCCAT-3′) was described and validated here [72]. Both morpholinos have a green-emitting fluorescent tag, 3′-carboxyfluorescein, and all morpholinos were synthesized in GeneTools, USA. After the injections, the embryos developed to NF stage 20 to analyse the NT closure. The phenotypes from different experiments were annotated and shown in a graph. Embryo images were acquired by Zeiss Axio Zoom V16 stereomicroscope and associated Zen software.

For immunostaining, the embryos were blocked for 1 h at room temperature in blocking buffer (5% animal serum, 1% BSA, 1% dimethyl sulfoxide (DMSO) in PBSTr 0.1) and incubated overnight with primary antibodies (1:300) (GFP SC B-2; pMLC pS20 Abcam, ab2480) at 4°C. The next day after washing, embryos were incubated overnight in 0.1% BSA, 1% DMSO and 0.1% PBSTr with secondary antibodies (1:300) (Alexa Fluor 488 Goat AntiMouse IgG (H+L), Invitrogen, A-11001—1:300; Cy3 AffiniPure Donkey Anti-Rabbit IgG (H+L), 711-165-152, Jackson ImmunoResearch—1:300) at 4°C. The next day after washing, the embryos were visualized using SIM to capture ectoderm or neuroectoderm images. For visualization of polarized neuroectoderm on confocal microscopy, a neural plate was cut and mounted by Vectashield Mounting Medium (H-1000, Baria).

For live imaging, *Xenopus* embryos were microinjected with 100 pg of plasmid DNA of Pk2 WT in the four-cell stage into two dorsal blastomeres. After the injections, the embryos were left to develop to NF stage 10.5 to observe the ectoderm. Then, they were embedded in Agarose Low Melt (6351.1, Roti). For live imaging, we used a fluorescence stereo zoom microscope (Zeiss AxioZoom.V16) equipped with AxioCam 512 mono monochromatic camera, PlanNeoFluar Z 2.3× objective, transmitted light source CL 9000 LED CAN 488 nm and brightfield with 100% intensity, with an exposure time of 100 ms, together with Zen Blue software. The frames were acquired each minute. Optical z-sections were separated by about 5 µm. Then the images were processed by function *Extended Depth of Focus* in Zen Blue software with default settings.

### 4.8. Confocal and structured illumination microscopy

For MDCK cell culture imaging, fluorescent confocal images were collected using a Leica SP8 microscope equipped with 63×/1.4 oil objective and LAX software (version 3.7.4). For the measurement of cell volume, a Leica SP8 microscope equipped with an HC PL APO CS2 40×/1.10 water objective was used. For calculation of cell volumes, the *Surface* module of Imaris 10.0.0 (Oxford Instruments, Abingdon, UK) was used with Object-Object Statistic and Segment Only the Region of Interest options, surface detail 1 μm.

For *Xenopus* embryos, a Zeiss Elyra 7 with Lattice SIM, equipped with C-Apochromat 40×/1.2 water objective and 2×PCO edge sCMOS camera, 488, 552 and 638 nm solid-state lasers with 0.2–1% intensity, exposure time 50 ms, was used together with Zen Black software. Optical z-sections were separated by about 20 µm. To create three-dimensional clusters of Pk2 and pMLC (figure 3*c′*), the *Surface* module of Imaris 9.9.1 (Oxford Instruments, Abingdon, UK) was used with default settings.

### 4.9. Numerical data and statistics

As for numerical data, mean  ±  s.d. are shown as depicted. For two-column statistics, an unpaired two-tailed *t*‐test was used. For cell volume statistics, an ordinary one-way ANOVA test was used (*p ≤ 0.05; **p ≤ 0.01; ***p ≤ 0.001; ****p ≤ 0.0001; ns, not significant, *p* > 0.05).

Graph of cell volumes was created using SuperPlotsOfData software (https://huygens.science.uva.nl/SuperPlotsOfData/) with mean and s.d. error bars. The ordinary one-way ANOVA with Tukey’s multiple comparisons test was performed by GraphPad Prism 9.

## Data Availability

For MS data evaluation, we used MaxQuant software (v2.4.2.0) [73] with the inbuilt Andromeda search engine [74]. Search was done against protein databases of Canis lupus (59 105 protein sequences, version from 2023-06-13, downloaded from https://www.uniprot.org/proteomes/UP000805418; including three custom proteins: GFP-xPrickle2, Flag-cMLC9, myc-xDvl2) and cRAP contaminants (112 sequences, version from 2018-11-22, downloaded from http://www.thegpm.org/crap). Modifications were set as follows for database search: oxidation (M), deamidation (N, Q) and acetylation (Protein N-term) as variable modifications, with carbamidomethylation (C) as a fixed modification. Enzyme specificity was tryptic with two permissible miscleavages. Only peptides and proteins with a false discovery rate threshold under 0.01 were considered. Relative protein abundance was assessed using protein intensities calculated by MaxQuant. Intensities of reported proteins were further evaluated using a software container environment (https://github.com/OmicsWorkflows/KNIME_docker_vnc; version 4.7.7a). Processing workflow is available upon request, and it covers, in short, reverse hits and contaminant protein groups (cRAP) removal, protein group intensities log2 transformation and normalization (loessF). MS proteomics data (figure 5*a*) were deposited to the ProteomeXchange Consortium via the PRIDE [75] partner repository under dataset identifier PXD052350. We utilized standard software such as Excel and GraphPad for generating the graphs, Fiji for signal profiling, Irfanview for imaging, and PowerPoint for figure layout creation. All figures in this research paper were designed with colourblind-friendly colours to ensure maximum accessibility. Supplementary material is available online [76].
